# Counting the Photons: Determining the Absolute Storage Capacity of Persistent Phosphors

**DOI:** 10.3390/ma10080867

**Published:** 2017-07-28

**Authors:** David Van der Heggen, Jonas J. Joos, Diana C. Rodríguez Burbano, John A. Capobianco, Philippe F. Smet

**Affiliations:** 1 Lumilab, Department of Solid State Sciences, Ghent University, Krijgslaan 281 S1, 9000 Gent, Belgium; david.vanderheggen@ugent.be (D.V.d.H.); jonas.joos@ugent.be (J.J.J.); 2 Department of Chemistry and Biochemistry and Center for Nanoscience Research, Concordia University, Montreal, QC H4B 1R6, Canada; di_rodr@live.concordia.ca (D.C.R.B.); John.Capobianco@concordia.ca (J.A.C.)

**Keywords:** persistent luminescence, storage capacity, strontium aluminate, calcium sulfide, rare earths

## Abstract

The performance of a persistent phosphor is often determined by comparing luminance decay curves, expressed in cd/m${}^{2}$. However, these photometric units do not enable a straightforward, objective comparison between different phosphors in terms of the total number of emitted photons, as these units are dependent on the emission spectrum of the phosphor. This may lead to incorrect conclusions regarding the storage capacity of the phosphor. An alternative and convenient technique of characterizing the performance of a phosphor was developed on the basis of the absolute storage capacity of phosphors. In this technique, the phosphor is incorporated in a transparent polymer and the measured afterglow is converted into an absolute number of emitted photons, effectively quantifying the amount of energy that can be stored in the material. This method was applied to the benchmark phosphor SrAl${}_{2}$O${}_{4}$:Eu,Dy and to the nano-sized phosphor CaS:Eu. The results indicated that only a fraction of the Eu ions (around 1.6% in the case of SrAl${}_{2}$O${}_{4}$:Eu,Dy) participated in the energy storage process, which is in line with earlier reports based on X-ray absorption spectroscopy. These findings imply that there is still a significant margin for improving the storage capacity of persistent phosphors.

## 1. Introduction

An important class of luminescent materials, usually referred to as persistent or afterglow phosphors, possess the capability to store energy for a period of time. This energy is stored during the charging of the phosphor, typically achieved by irradiation with blue or near-UV (ultraviolet) light, after which it is gradually released, manifesting itself as delayed emission. The field of persistent luminescent materials gained importance with the discovery of SrAl${}_{2}$O${}_{4}$:Eu,Dy by Matsuzawa et al. [1], replacing ZnS:Cu,Co [2] as the benchmark afterglow phosphor. This green emitting phosphor shows afterglow for several hours after the excitation has stopped, and has been extensively investigated [3,4,5]. Two decades after the discovery by Matsuzawa et al., the list of afterglow phosphors has grown, and in addition to the alkaline earth aluminates it now also includes, among others, silicates and sulfides doped with a wide variety of activators, such as Eu${}^{2+}$ [6], Ce${}^{3+}$ [7,8] or transition metal ions [9]. An important class of these materials are the red- and infrared-emitting persistent phosphors, which are quite scarce compared to other classes, but are highly desired in diverse applications, such as bioimaging and safety signage. These phosphors are expected to exhibit efficient afterglow and are often based on the luminescence of transition metal ions [10,11,12].

A well-studied, red-emitting phosphor that is not based on transitions metals is CaS:Eu. Although this sulfide, like many other sulfides, has a relatively poor chemical stability, its high luminescence efficiency and the limited broadness of the emission spectrum led researchers to suggest that this phosphor could be applied as a white LED (light-emitting diode) phosphor. This claim was further supported by the discovery of the color tunability of the Eu${}^{2+}$ emission in the Ca${}_{x}$Sr${}_{1-x}$S solid solution [13]. It was quickly found that the phosphor exhibits persistent luminescence when it is codoped with other lanthanides [14,15], which possibly paves the way for other applications. Recently it was reported that the persistent luminescence also arises in CaS:Eu if the grain size of the powder is reduced to a few hundred nanometers [16]. The dimensions of the grains in the nano-sized regime, in combination with the spectral location of the emission, provides the phosphor with characteristics for applications such as optical imaging in biomedicine. However, the surface must be functionalized in order to render the nanoparticles stable in aqueous media [17,18,19].

The performance of persistent phosphors is often determined on the basis of luminance decay curves, which are usually measured for as long as the emission can be perceived by the dark-adapted eye [20]. Although this approach provides a relatively easy way to compare different persistent phosphors, the method also entails some problems.

Commonly, decay curves are presented in arbitrary units [21,22], allowing only for a comparison with other phosphors presented within the same work. Moreover, even if the decay curves are presented in the correct photometric units, a direct comparison concerning the trapping capabilities of different phosphors is not possible for reasons that are inherent to luminances and the way they are measured. A photometric measure such as the luminance depends strongly on the spectral shape of the emission, and, more specifically, on how it overlaps with the spectral eye sensitivity. This implies that two phosphors with a comparable storage capacity but a different emission spectrum can have very different luminances [23]. An extreme example is the infrared persistent phosphors, for which the emission does not overlap with the eye sensitivity curve; therefore they have a zero luminance by definition. Another drawback, which is often overlooked, originates from the fact that luminance measurements are usually performed on an infinitely thick sample, ensuring that the layer thickness of the powder is higher than the penetration depth of the excitation light. Although this approach guarantees that measurements on the same material are reproducible, it inhibits a meaningful comparison between different materials because variations in the penetration depth of the excitation light in different materials will lead to different amounts of phosphor being excited.

Transparent samples, such as ceramic or glass-ceramic phosphors, are notable examples for which a seemingly better performance in terms of persistent luminescence is achieved due to a marked increase in the penetration depth of light compared to polycrystalline powders. Typical examples are transparent ceramics of Cr${}^{3+}$ or Ce${}^{3+}$ activated garnets [24,25,26], (glass-)ceramics of SrAl${}_{2}$O${}_{4}$:Eu${}^{2+}$, Dy${}^{3+}$ [27,28,29], near-infrared-emitting ZnGa${}_{2}$O${}_{4}$:Cr${}^{3+}$ [30,31] and other oxide phosphors [10,32,33]. The luminance or radiance decay curve of a thick ceramic sample will remain above a pre-defined threshold for a much longer time than for a powder sample with a comparable storage capacity and trap depth distribution, making an objective comparison between powder and ceramic samples impossible unless the amount of excited phosphor material is well-controlled.

**Figure 1:**
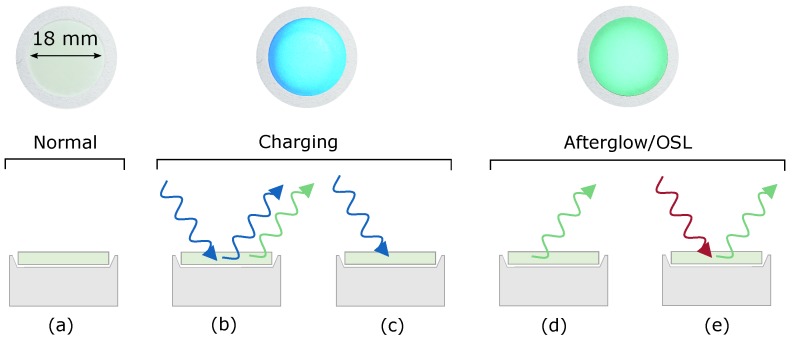
Schematic of the experimental procedure to measure the absolute storage capacity of phosphors. The top of each panel shows a photograph of a polymer layer on the sample holder (top view) under different experimental conditions. The bottom part of each panel represents a schematic side view of the sample shown in the top part. (**a**) An uncharged polymer layer; (**b**) Reflection of the excitation light (blue) and absorption leading to photoluminescence (green); (**c**) Absorption of excitation light (blue) leading to trapping; (**d**) Spontaneous afterglow (green) after excitation; (**e**) Absorption of a (low-energy) photon (red) leading to the release of a trapped charge and yielding emission of light (green). This effect is called optically stimulated luminescence (OSL).

One way to overcome this drawback is to deposit the phosphor as a thin film with a thickness that is less than the penetration depth of the excitation light in that material. However, another approach that requires less-elaborate experimental conditions is to incorporate a well-known amount of the phosphor in a transparent polymer. This ensures that the luminescent material is sufficiently spread out, such that a homogeneous excitation of the phosphor is accomplished. The determination of the absolute storage capacity of the phosphor can then simply be accomplished by measuring the afterglow of this polymer layer and converting the decay profile into an absolute number of photons. This latter approach is used to estimate the amount of energy that can be stored in SrAl${}_{2}$O${}_{4}$:Eu,Dy and CaS:Eu nanoparticles. Figure 1 shows a schematic overview of the developed method to measure the absolute storage capacity of the phosphors in the polymer layer.

The storage capacity that is obtained in this way only takes into account the emptying of shallow traps that are responsible for the afterglow exhibited by the phosphors at room temperature. Deep traps that are not emptied at room temperature are therefore not taken into account. However, for many applications, such as safety signage, traffic markings [34,35], optical memory applications [36] or bio-imaging [17,37,38,39], these are exactly the traps that are considered to be useful. The use of external stimuli, such as temperature and infrared light, can, however, offer a way to expand this method to determine the full storage capacity of these phosphors. An accessible way to determine the total trapping capacity is needed, as it is an important property that defines the usability of the phosphors in many applications. In many cases, further improvement is still needed, which could, for example, lead to highly demanding applications such as traffic markings that emit light overnight, or bio-imaging phosphors that do not have the need to be recharged after injection [40,41].

## 2. Materials and Methods

The measurements have been performed on commercial SrAl${}_{2}O_{4}$:Eu,Dy (GloTech Intl.; Auckland, New Zealand; GT 8400, 0.88% Eu and 1.6% Dy) and on nano-sized CaS:Eu synthesized by the co-precipitation method. The phase purity of both phosphors was verified by powder X-ray (Cu Kα) diffraction measurements using a Siemens D5000 diffractometer. Details concerning the synthesis, characterization and structural properties of the CaS:Eu nanophosphor were described by Rodríguez Burbano et al. [16]. The phosphors were incorporated into a polymer layer by mixing an appropriate amount of the powders in a 20 wt% solution of kraton FG1901X in 3:1 toluene-to-methyl ethyl ketone, after which the mixture was cast on a circular glass plate with a diameter of 18 mm [42]. Care was taken to avoid the inclusion of air bubbles and to obtain a homogeneous phosphor loading over the glass plate area. After evaporation of the solvents at room temperature, a uniform phosphor-containing polymer layer was obtained (Figure 1a (top)).

Photoluminescence emission spectra were recorded using a ProEM1600 EMCCD camera attached to an Acton SP2300 monochromator (Princeton Instruments; Trenton, NJ, USA), which was intensity calibrated using a DH-2000-CAL calibration lamp (Ocean Optics; Dunedin, FL, USA). Afterglow measurements were performed using an ILT 1700 calibrated photometer (International Light Technologies; Peabody, MA, USA) equipped with a photopic filter (YPM). The samples were placed on a diffuse reflective BaSO${}_{4}$-coated sample holder to enable detection of the light emitted in all directions. SrAl${}_{2}O_{4}$:Eu,Dy and CaS:Eu were excited using LEDs with a wavelength of 450 and 470 nm, respectively. A high-power infrared LED with a wavelength of 970 nm was used to induce optically stimulated luminescence (OSL; see Figure 1e) in CaS:Eu, and a low-pass filter ($\lambda_{c}=700$ nm) was used to block the stimulating light. Diffuse transmission spectra were recorded using a Varian Cary 500 UV-VIS-NIR spectrophotometer equipped with a 110 mm BaSO${}_{4}$-coated integrating sphere.

## 3. Results

The photometric data from a luminance decay curve can be converted into an absolute number of photons by using Equation (1), whereby one converts the luminance reading to a photon stream by taking the luminance efficacy and the average photon energy into account and integrating over all emission angles:

(1) $$\begin{array}{c} N_{\gamma }=\;\int \int \;\frac{L(t,\theta )A}{\eta {\bar{E}}_{\gamma }}d\Omega dt \end{array}$$

Here, L(t,θ) is the luminance as a function of time and solid angle in cd/m${}^{2}$, *A* is the surface area of the sample, η is the luminous efficacy of the emission spectrum in lm/W and ${\bar{E}}_{\gamma }$ is the average photon energy of the emission spectrum of the phosphor. It can readily be seen from this equation that information on the angular dependence of the emission is needed to perform the integration over the solid angle. In general, as a first approximation, flat and uniform surfaces such as the polymer layers may be considered to be Lambertian emitters. However, as it will be discussed later, a more accurate result can be obtained if the angular emission profile is measured.

Moreover, in addition to the information regarding the angular dependence of the emission, an emission spectrum is needed to determine the luminous efficacy and the average photon energy. The spectrum must be corrected for the instrument response to yield a photon flux per wavelength interval $\phi_{\gamma }\left(\lambda \right)$ [43]. The luminous efficacy and the average photon energy can then be determined using Equations (2) and (3), in combination with the known eye sensitivity curve V(λ) [23]:

(2) $$\eta =\frac{\int \phi_{e}\left(\lambda \right)V\left(\lambda \right)d\lambda }{\int \phi_{e}\left(\lambda \right)d\lambda }$$

(3) $${\bar{E}}_{\gamma }=\frac{\int \phi_{\gamma }\left(E\right)EdE}{\int \phi_{\gamma }\left(E\right)dE}$$

where $\phi_{e}\left(\lambda \right)$ is the spectral radiant flux, which is related to the photon flux by $\phi_{e}\left(\lambda \right)$d$\lambda =\phi_{\gamma }\left(\lambda \right)\frac{hc}{\lambda }$dλ. Care should be taken when converting a flux per wavelength interval into a flux per energy interval, as the relation in Equation (4) holds [44]:

(4) $$\frac{d\phi (E)}{dE}∼\lambda^{2}\frac{d\phi (\lambda )}{d\lambda }$$

The method outlined in this section was validated using the commercial SrAl${}_{2}$O${}_{4}$:Eu,Dy phosphor and was then applied to the CaS:Eu nanophosphor to demonstrate its versatility in determining the absolute storage capacity with relatively low experimental effort.

### 3.1. Validating the Method: SrAl${}_{2}$O${}_{4}$:Eu,Dy

**Figure 2:**
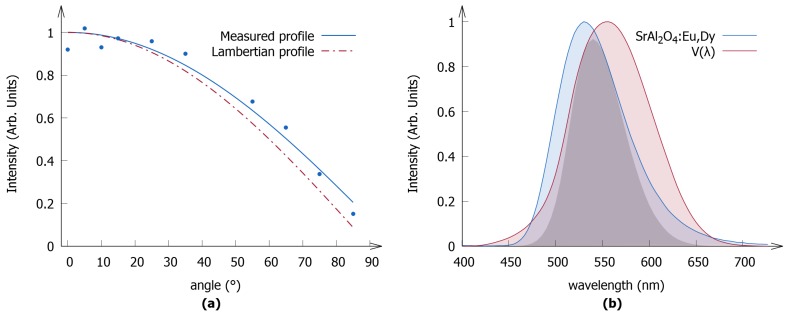
(**a**) Angular distribution of the emission of the polymer layers; the Lambertian profile is added as a reference; (**b**) The emission spectrum (photon flux) of SrAl${}_{2}$O${}_{4}$:Eu,Dy is shown in blue. The spectrum was recorded at 300 K under excitation at 400 nm. The photopic eye sensitivity curve is shown in red. The product of both curves is depicted as the shaded gray area.

Figure 2a shows the angular distribution emission profile of the SrAl${}_{2}$O${}_{4}$:Eu,Dy polymer layer and how it deviates from a Lambertian profile. The deviation, most evident at larger angles, may be attributed to the specific geometry of the sample holder, which, in this case, redistributes the intensity to larger angles. This illustrates the need to measure the angular dependence of the emission as the initial approximation of the layers, as Lambertian emitters would have led to an underestimation of the storage capacity.

The emission spectrum of SrAl${}_{2}$O${}_{4}$:Eu,Dy is shown in Figure 2b. It exhibits the characteristic broadband emission of Eu${}^{2+}$, which has a maximum at around 530 nm attributable to the radiative transition from the lowest 4f${}^{6}$5d${}^{1}$ excited state to the 4f${}^{7}$ ground state [45]. The emission spectrum overlaps fairly well with the eye sensitivity curve, which results in a high luminous efficacy of 477.80 lm/W. The average photon energy was found to be 2.25 eV.

**Figure 3:**
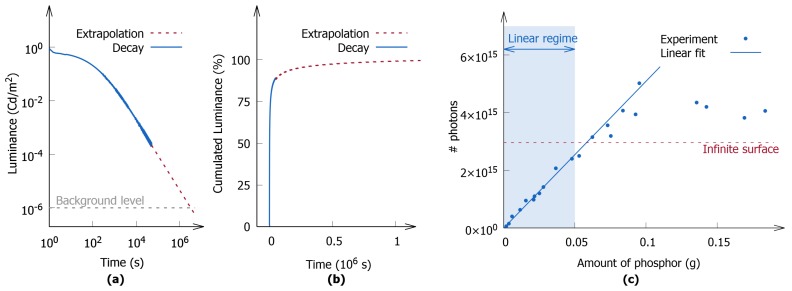
(**a**) An afterglow curve for a polymer layer containing 0.0032 g of SrAl${}_{2}$O${}_{4}$:Eu,Dy. The extrapolation method and the instrumental background level are also indicated; (**b**) Cumulative decay curve indicating the fractions of the total luminance that are measured or deduced from extrapolation; (**c**) The number of photons that were released from SrAl${}_{2}$O${}_{4}$:Eu,Dy during the afterglow after 15 min of excitation with a 450 nm LED ($E_{e}=0.43$ mW/cm${}^{2}$). The region where the number of photons was linearly related to the amount of phosphor incorporated in the layers is also indicated. The level indicated as “infinite surface” corresponds to the number of photons calculated from a luminance decay curve that was measured on an infinitely thick surface.

The storage capacity of SrAl${}_{2}O_{4}$:Eu,Dy was determined by preparing multiple polymer layers with different amounts of phosphor, ranging from 0.0018 to 0.1850 g. All the layers were charged using a 450 nm LED with a constant irradiance of 0.43 mW/cm${}^{2}$ over 15 min, a time that was sufficiently long to arrive at a dynamic equilibrium between the storage and release of energy. After charging, the afterglow decay curves were recorded. An example of such a decay curve is shown in Figure 3a, where the characteristic long-persistent luminescence duration of up to several hours is exhibited. To minimize the time length for the acquisition of data, the afterglow was measured for several hours. Then, the final portion of the decay profile was extrapolated to the background level. The cumulative luminance is represented on a linear scale in Figure 3b, and the stagnating behavior of the curve justifies the approach outlined above. The figure also illustrates that a measurement of several hours already covers a large fraction of the total emission, as the first 65% of the total intensity was recorded during the first hour of afterglow, and this fraction could have been increased to 80% if the measurement duration was extended to 10 h.

Following the acquisition of data, the afterglow decay curves were converted to an absolute number of photons using the aforementioned method, and the results are shown in Figure 3c. Two regions were identified on the basis of how the luminescent output was related to the amount of phosphor integrated in the polymer layer.

The first region is characterized by a linear dependence of the optical output on the incorporated amount of phosphor, and is situated in the part of the curve that contains polymer layers with low loading. This region was used to determine the absolute storage capacity of the phosphor, resulting in a storage capacity of $\left(5.1\pm 0.2\right)\times 10^{16}$ photons per gram of material. The remainder of the graph is characterized by an initial stagnation, followed by a slight decrease in the luminescent output when elevating the amounts of phosphor, and cannot be used to determine the storage capacity, as it is unclear how much of the phosphor was exposed to the excitation light (see Section 3.1.2).

#### 3.1.1. Intensity Dependence

**Figure 4:**
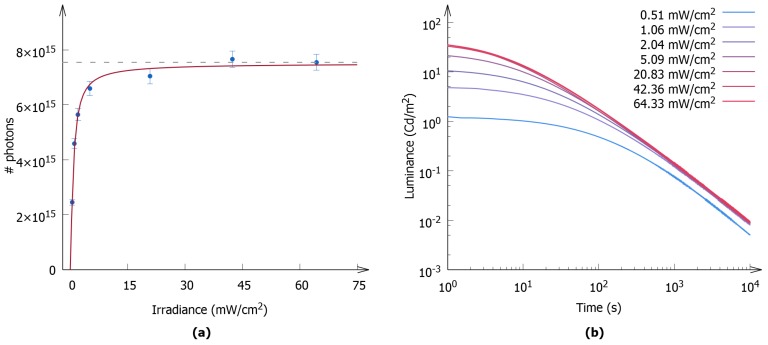
(**a**) The measured storage capacity for SrAl${}_{2}$O${}_{4}$:Eu,Dy (blue dots) as a function of the irradiance of the excitation light during charging. The first data point, at the lowest excitation intensity, corresponds to the experimental conditions used for the experiments shown in Figure 3. The red line is a guide for the eye; (**b**) The decay curves for different excitation intensities.

The measurements described above have been performed for different loadings of the polymer layers, keeping the irradiance of the excitation light constant for all the samples. One could question whether the storage capacity would change if the intensity of the excitation light is varied. Therefore the storage capacity was measured as a function of the excitation intensity for one polymer layer with a loading of 48 mg of SrAl${}_{2}$O${}_{4}$:Eu,Dy. The results are shown in Figure 4a, and they reveal an increase in the storage capacity as the intensity increased. A linear increase is observed for low irradiance, whereas at an irradiance higher than 5 mW/cm${}^{2}$ a plateau is obtained, indicating an absolute trapping capacity had been reached. The absolute and maximal storage capacity of this polymer layer was found to be $\left(7.51\pm 0.13\right)\times 10^{15}$, corresponding to a storage capacity of $\left(1.57\pm 0.03\right)\times 10^{17}$ photons per gram of SrAl${}_{2}$O${}_{4}$:Eu,Dy.

The luminance decay curves were measured as a function of the excitation intensity, and are shown in Figure 4b. As the excitation intensity increases, the luminance decay curve shifts to higher luminance values. However, at higher values of the excitation intensity, the shift is less significant—it is almost unchanged. At values greater than 42.36 mW/cm${}^{2}$, no changes in the decay curve are observed, allowing us to state that an absolute, maximum storage had been reached.

#### 3.1.2. Defining the Boundaries of the Linear Regime

The nonlinear region observed in Figure 3c does not contain useful information to determine the storage capacity of phosphors. However, the information provided by this nonlinear regime can be used to establish the boundaries of the linear regime. In addition, this region can lead to the specification of the requirements to which a polymer layer has to comply in order to be used to determine storage capacities, which can considerably lower the experimental effort.

**Figure 5:**
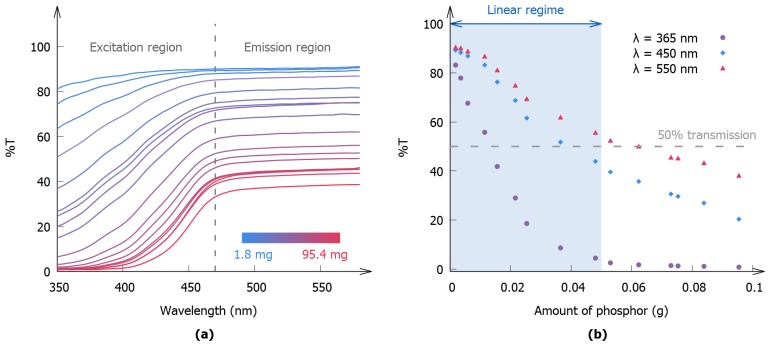
(**a**) Diffuse transmission spectra of polymer layers with different amounts of SrAl${}_{2}$O${}_{4}$:Eu,Dy incorporated. The plot is divided into a low- and a high-wavelength range, covering the excitation and emission wavelengths of the phosphor, respectively; (**b**) The transmission of the layers at 365 nm, at the excitation wavelength used in this work (450 nm), and at the emission wavelength (550 nm). The linear regime from Figure 3 is indicated as a reference.

To gain further insight into the processes that lie at the origin of this nonlinear behavior, it is imperative to study the diffuse transmission of the phosphor layers. The transmission spectra of the individual polymer layers are represented in Figure 5a. A distinction is made between an excitation region, which is characterized by an absorption band that originates from the 4f${}^{7}\to$ 4f${}^{6}$5d${}^{1}$ transitions of Eu${}^{2+}$ [45], and an emission region that covers the wavelength range where the emission of the phosphor is located.

The transmission of the excitation light poses a limit on the amount of phosphor in the layer that can be exposed to the excitation light. If too much material is added to the layer, shadowing effects will arise and the excess powder will not be excited. This will result in a stagnation of the optical output as a function of the incorporated amount of phosphor. On the other hand, the transmission at the emission wavelength will determine how much of the initially emitted light will reach the detector. If the layer becomes too opaque, light that is reflected off the sample holder will not be detected, and only half of the emitted light will be recorded. This effect will give rise to a decrease in the optical output with an increase of phosphor content. The combination of these effects gives rise to the nonlinear behavior illustrated in Figure 3c. In an attempt to quantify these two effects, the transmission at the excitation and emission wavelengths is represented in Figure 5b. It is apparent from this figure that all polymer layers in the linear regime had an average transmission for excitation and emission light that lies above 50%. We therefore propose to use this criterion as a rule of thumb to discriminate between layers that are situated in the linear regime and layers that are not.

The transmission of the layers at 365 nm, a frequently used excitation wavelength, is shown in the same figure. It can readily be seen that the transmission quickly decreases with an increasing loading. This is due to the fact that this wavelength is located near the center of the excitation band leading to high absorption. Applying the rule of thumb to this curve leads to the conclusion that the range of loadings for which a layer ends up in the linear regime drastically decreases. It can thus be concluded that it is desirable to excite the phosphor near the edge of an absorption band.

### 3.2. Applying the Method: CaS:Eu

**Figure 6:**
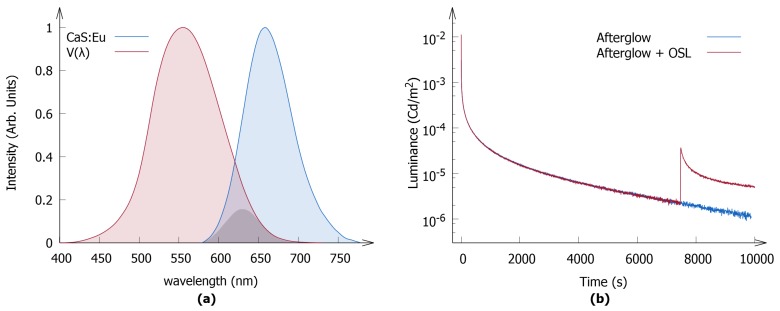
(**a**) The emission spectrum (photon flux) of CaS:Eu is shown in blue. The spectrum was recorded at 300 K under excitation at 475 nm. The photopic eye sensitivity curve is shown in red. The product of both curves is depicted as the shaded gray area; (**b**) Luminance decay profiles of the polymer layer containing 9 mg of the CaS:Eu nanophosphor after excitation with a 475 nm LED. The OSL is clearly visible as a rise in intensity upon continuous irradiation of the sample with infrared light (λ=970 nm).

The validated method was applied to study the CaS:Eu nanophosphor. The emission spectrum is shown in Figure 6a and exists as a single emission band peaking at 657 nm (full width at half maximum of 80 nm). The measurements for this phosphor have been performed on a single polymer layer containing 9 mg of the nanopowder. The afterglow was measured after exciting the phosphor for 15 min with a 475 nm LED, which corresponds to excitation into the first 4f${}^{7}\to 4$f${}^{6}$5d${}^{1}$ band of Eu${}^{2+}$ in CaS. These measurements resulted in a storage capacity of $\left(7.3\pm 0.3\right)\times 10^{14}$ photons per gram.

Recently, Rodríguez Burbano et al. reported that this material also exhibits OSL under illumination with infrared light [16]. Therefore, the sample was illuminated with 970 nm infrared light after measuring the initial afterglow. As can be seen from Figure 6b, this indeed results in OSL, which manifests itself as a regeneration of the decay curve. If the additional optical output is taken into account, the storage capacity of the phosphor nearly doubles, to about $\left(12.2\pm 0.5\right)\times 10^{14}$ photons per gram of phosphor, which corresponds to an average of about 100 photons per nanoparticle.

## 4. Discussion

The storage capacity of a phosphor, as it was determined for SrAl${}_{2}$O${}_{4}$:Eu,Dy in the previous section, does not correspond to a general, overall maximum storage capacity of the phosphor. It is rather the result of several cooperating processes that together determine the storage capacity of the phosphor under specific experimental conditions, such as the temperature and excitation wavelength [45]. Among these processes, OSL by the excitation light during charging can have a major impact on the storage capacity of a phosphor. This effect was recently reported for Sr${}_{2}$MgSi${}_{2}$O${}_{7}$:Eu,Dy [46].

Moreover, it is important to consider possible non-radiative losses, as not every released charge will lead to a radiative decay. A quantity taking this into account is the afterglow quantum efficiency, which is defined as the efficiency by which the release of a trapped charge leads to the emission of a photon. Botterman et al. [45] estimated the afterglow quantum efficiency of SrAl${}_{2}$O${}_{4}$:Eu,Dy to be around (65 ± 10)%. They also found that the light output, measured during the first 45 min of afterglow at room temperature, can be doubled by raising the temperature during a subsequent thermoluminescence (TL) experiment. It is worth noticing that also in CaS:Eu, the estimated storage capacity is doubled when applying external infrared light photo-stimulation in order to empty deeper traps that are not accessible at room temperature.

Various theoretical models attempt to describe the trapping mechanism in persistent phosphors [47,48,49] and despite that the chemical nature of the traps in these models is different or often not specified, they all suggest that the trapped charges originate from photo-ionized Eu${}^{2+}$ centers. Considering the quantum efficiency of the afterglow and doubling the light output measured in the first 45 min, to take into account the amount of traps that can additionally be emptied during TL, it can be concluded that after charging, 1 g of material contains about $5\times 10^{17}$ filled traps. This roughly corresponds to about 1.6% of the Eu${}^{2+}$ ions participating in the energy storage process. Korthout et al. [50] reported changes in the XANES (X-ray Absorption Near Edge Spectroscopy) signal of a few percent upon charging SrAl${}_{2}$O${}_{4}$:Eu,Dy, indicating a valence change of a similar fraction of the Eu ions within the material, which is in agreement with the estimation above.

The storage capacity of SrAl${}_{2}$O${}_{4}$:Eu,Dy and CaS:Eu can be compared to the storage capacity of LiYSiO${}_{4}$:Ce (5%) [51]. This is one of the many materials that are reported in literature as storage phosphors. The penetration depth of the α-radiation, which is used as an excitation source, was calculated to be considerably less than the penetration depth of the stimulation light used to empty the traps. Based on the information provided by Knitel et al., LiYSiO${}_{4}$:Ce exhibits a storage capacity of about 10${}^{13}$ photons per gram after exposure of the material to 1.7 μGy of α-radiation. It is important to note that despite the vast number of reports on storage phosphors, only a few contain enough information to determine the storage capacity, and even less describe samples that were prepared according to the requirements stated above.

Every measurement method inevitably comes with its own limitations, and the method described above is of course no exception. The incorporation of the phosphor into the polymer matrix has consequences for the optical properties. Phosphors are often characterized by a high refractive index (approximately 1.6 and 2.1 for SrAl${}_{2}$O${}_{4}$:Eu,Dy and CaS:Eu, respectively [52,53]), whereas polymers generally possess a low refractive index (1.51 for kraton FG1901X [54]). This significant difference will have an effect on optical properties, such as outcoupling and the scattering of light in the polymer layers, and will introduce an uncertainty into the obtained results. The latter effect mainly plays an important role during the excitation, as scattering determines how much material can be exposed to the excitation light. The boundaries of the linear regime will thus, to some extent, depend on the refractive indices of the materials. Moreover, the scattering properties are influenced by the morphology and the dimensions of the phosphor grains, which will have a moderate effect on the measured storage capacities. Although these effects are important, they are expected to have only a moderate impact on the determined storage capacities [52].

**Figure 7:**
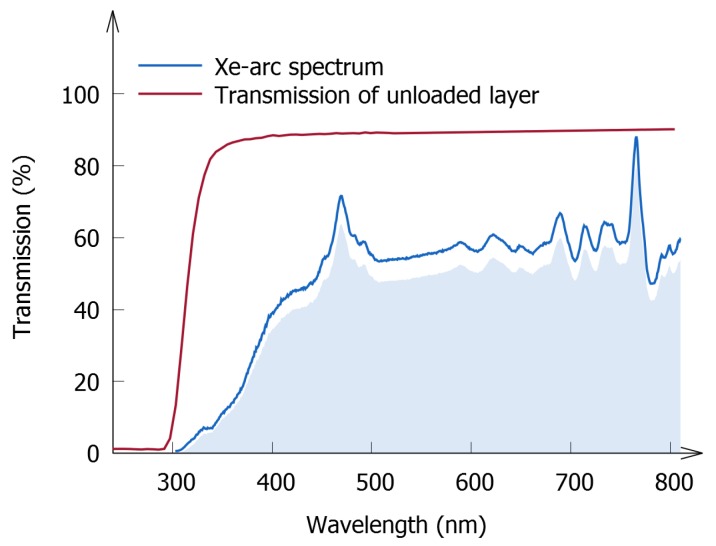
Diffuse transmission spectrum of an unloaded (i.e., without-phosphor) polymer layer. The spectrum of the 1000 lux Xe-arc industrial standard is also depicted. The shaded area indicates the fraction of light that can be used for excitation of the phosphor, taking into account losses due to the glass substrate and polymer.

Finally, we evaluate to what extent the developed method can be incorporated in already established measuring methods used in the industry, in which a 1000 lux Xe-arc lamp is required for excitation. To that purpose, the transmission of an unloaded polymer layer was measured; it is presented in Figure 7 and compared with the spectrum of the Xe-arc lamp. The absorption edge of the unloaded sample is located at around 300 nm, which is before the major onset of the spectrum of the lamp, suggesting that a substantial part of the light emitted by the lamp can indeed be used to excite the phosphor. This means that the industry-standard excitation source can be combined with the method described above. However, care should be taken, as other polymers might have a higher absorption. Moreover, many non-Eu${}^{2+}$-based phosphors, such as transition metal-activated phosphors, need to be excited by deep UV light, which might pose problems, considering the above methodology. In these cases, it is important to check the optical compatibility of the used polymer, which can be done by standard UV-vis absorption spectroscopy.

Furthermore, we would like to express a concern related to the use of the 1000 lux Xe-arc lamp as an excitation source to measure storage capacities. The Xe-arc lamp has a considerable optical output in the infrared, which will induce OSL while charging, thereby limiting the storage of energy during the first stages of the experiment. Moreover, it can be argued that the spectrum of the lamp, which reproduces daylight conditions fairly well, is a poor representation of the indoor conditions under which many of the envisaged applications take place. Lastly, it is important to consider that the wavelength dependence of the transmission, as discussed in Section 3.1.2, might introduce problems when using a white excitation source. Considering all of the above, we would propose the use of a more monochromatic excitation source, such as those used in this work.

## 5. Conclusions

A method to determine the absolute storage capacity of a persistent phosphor from its luminance decay curve was introduced. This method enables an unambiguous comparison between the performance of different persistent phosphors, and was validated using commercial SrAl${}_{2}$O${}_{4}$:Eu,Dy. The storage capacity of this benchmark phosphor was found to be equal to $\left(1.57\pm 0.03\right)\times 10^{17}$ photons per gram of SrAl${}_{2}$O${}_{4}$:Eu,Dy. These results imply that only a small fraction of the Eu ions participate in the storage process, which agrees with earlier results based on XANES measurements. Subsequently, this method was applied to red-emitting nano-sized CaS:Eu, illustrating that the method indeed enables the comparison of persistent phosphors with very different emission spectra. The storage capacity of the nanophosphor was found to be $\left(12.2\pm 0.5\right)\times 10^{14}$ photons per gram of CaS:Eu, or about 100 photons per nanoparticle, when deeper traps, accessible by infrared stimulation, are included. This method now allows us to evaluate unambiguously the influence of (nano)particle size and surface chemistry on the absolute trapping capacity of a particular phosphor, as compared to the bulk counterpart.

Finally, it was evaluated whether the Xe-arc lamp, which is considered to be an industrial standard, can be used as an excitation source. It was found that there are no practical limitations to combine the Xe-arc lamp with the proposed measurement method. Nevertheless, some compelling arguments were put forward that plead for the use of a more monochromatic light source when determining the storage capacity of a phosphor.
